# The Influence of Equipment Design and Process Parameters on Granule Breakage in a Semi-Continuous Fluid Bed Dryer after Continuous Twin-Screw Wet Granulation

**DOI:** 10.3390/pharmaceutics13020293

**Published:** 2021-02-23

**Authors:** Alexander Ryckaert, Michael Ghijs, Christoph Portier, Dejan Djuric, Adrian Funke, Chris Vervaet, Thomas De Beer

**Affiliations:** 1 Laboratory of Pharmaceutical Process Analytical Technology, Department of Pharmaceutical Analysis, Ghent University, Ottergemsesteenweg 460, 9000 Ghent, Belgium; AlexanderJ.Ryckaert@UGent.be; 2 BIOMATH, Department of Mathematical Modelling, Statistics and Bio-informatics, Ghent University, Coupure Links 653, 9000 Ghent, Belgium; Michael.Ghijs@UGent.be; 3 Laboratory of Pharmaceutical Technology, Department of Pharmaceutics, Ghent University, Ottergemsesteenweg 460, 9000 Ghent, Belgium; Christoph.Portier@UGent.be (C.P.); Chris.Vervaet@Ugent.be (C.V.); 4 Chemical & Pharmaceutical Development, Pharma R&D, Bayer AG, Friedrich-Ebert-Straße 475, 42117 Wuppertal, Germany; Dejan.Djuric@Bayer.com (D.D.); Adrian.Funke@Bayer.com (A.F.)

**Keywords:** continuous manufacturing, fluid bed drying, twin-screw granulation, process understanding, particle size distribution, moisture content, granules

## Abstract

The drying unit of a continuous from-powder-to-tablet manufacturing line based on twin-screw granulation (TSG) is a crucial intermediate process step to achieve the desired tablet quality. Understanding the size reduction of pharmaceutical granules before, during, and after the fluid bed drying process is, however, still lacking. A first major goal was to investigate the breakage and attrition phenomena during transport of wet and dry granules, the filling phase, and drying phase on a ConsiGma-25 system (C25). Pneumatic transport of the wet granules after TSG towards the dryer induced extensive breakage, whereas the turbulent filling and drying phase of the drying cells caused rather moderate breakage and attrition. Subsequently, the dry transfer line was responsible for additional extensive breakage and attrition. The second major goal was to compare the influence of drying air temperature and drying time on granule size and moisture content for granules processed with a commercial-scale ConsiGma-25 system and with the R&D-scale ConsiGma-1 (C1) system. Generally, the granule quality obtained after drying with C1 was not predictive for the C25, making it challenging during process development with the C1 to obtain representative granules for the C25.

## 1. Introduction

Among the different techniques for continuous pharmaceutical manufacturing of solid-dosage forms, continuous direct compression (CDC) is the most preferred technique if the involved material and formulation properties allow CDC [1,2,3]. As CDC only involves continuous feeding and blending of the raw materials, followed by tableting of the homogeneous powder blend, the number of intermediate process steps is limited [4]. However, CDC is not always applicable due to unfavorable material properties of the active product ingredient (API), such as poor flowability and compatibility and high electrostatics. Obviously, this is even more pronounced for high-loaded formulations [1,3]. In addition, low-dose formulations are also often impacted by poorly flowing APIs making it extremely challenging to homogenously disperse such API in the formulation powder blend. Consequently, tablet content uniformity might be impaired [1]. An intermediate granulation step such as roller compaction or twin-screw granulation (TSG) may be required to overcome aforementioned issues [5].

In the last 15 years, continuous twin-screw wet granulation has gained an increased interest within the pharmaceutical industry [6,7]. Many investigations have already been performed on continuous twin-screw wet granulation as unit operation. Some studies have been focusing on the influence of process parameters [6,8,9,10] and formulation properties [11,12,13,14,15,16,17,18] upon granule quality attributes. Other studies targeted a more fundamental understanding of the twin-screw granulation mechanism [7,8,19,20,21,22,23]. Many of these studies used the granulation module of a continuous from-powder-to-tablet line—the ConsiGma system (GEA Pharma systems, Wommelgem, Belgium) [7,8,9,11,12,13,14,19]. The six-segmented fluid bed drying following the granulation unit of the ConsiGma system has been investigated to a lesser extent, although the drying process is crucial to guarantee a good final product.

The stability and repeatability during a long production run of 5 h was evaluated using 1 formulation for the twin-screw granulation and six-segmented drying unit by Vercruysse et al. [24]. The study showed that the residual moisture content after drying remained stable during the complete run, indicating a reliable drying process for the formulation under study. Additionally, Vercruysse et al. [25] evaluated whether the product quality (i.e., residual moisture content, particle size distribution, bulk and tapped density, and friability) obtained by using only a single cell of the segmented dryer unit of the ConsiGma-25 system (C25) was comparable to the granule quality of granules collected during full-scale manufacturing when all drying cells were filled. As the granule quality was indeed similar, the use of a single cell could therefore be favorable during formulation and process development. However, the study did not investigate potential effects of varying drying parameters. In addition, the granule quality for an identical drying experiment (i.e., identical drying parameters) performed with a mobile ConsiGma-1 system (designed for R&D studies) was compared to the quality obtained during full-scale manufacturing with the ConsiGma-25 system. A deviating granule quality was observed, indicating that the ConsiGma-1 (C1) system was not predictive for the granule quality at steady state phase during full-scale manufacturing.

Other studies aimed at determining the granule moisture content by in-line near infrared (NIR) measurements in the ConsiGma-25 dryer [26,27]. In a recent study, Stauffer et al. [28] highlighted the influence of wet granule properties on drying stability (i.e., air flow deviations). Their study emphasized the importance of granule properties prior to drying as an excess of fine particles accumulating on the surface of the drying filter resulted in an unstable drying process. Moreover, an extensive investigation on the influence of drying process parameters on granule quality attributes and breakage behavior was performed by De Leersnyder et al. [29]. Both a horizontal and vertical set-up of the ConsiGma-25 was used in this study. The granulator is positioned next to the dryer in the horizontal set-up with a pneumatic granule transfer via a wet transfer line, whereas the granulator is positioned above the dryer with a gravimetric transfer of wet granules to the dryer in a vertical set-up. However, none of these aforementioned studies explored the evolution of the granule size between the granulator outlet and the conditioning unit. In addition, a one-on-one comparison of the drying behavior and granule quality between ConsiGma-1 and ConsiGma-25 had also never been performed at varying drying settings. However, it would facilitate the transfer from R&D equipment (ConsiGma-1) to clinical and commercial manufacturing (ConsiGma-25).

In the present study, granules were collected after the granulation module, after the wet transfer line connecting the granulator and dryer, in each off the drying cells (i.e., at different drying times) and after the dry transfer line connecting the dryer with the mill. This allowed us to evaluate the granule size distribution at each location throughout the process, whereby the degree of breakage and attrition could be attributed to each individual unit of a horizontal ConsiGma-25 line. Hence, this study was designed to fundamentally understand the impact of each process part on granule size. Moreover, these experimental results are essential in the development of a general flowsheet model as well as for a general drying model for the drying-unit of the ConsiGma-25 line. In addition, the current study also focused on a more in-depth comparison between the ConsiGma-1 and ConsiGma-25. Therefore, the effect of different drying parameters on final granule size, moisture content per size fraction, and overall moisture content was investigated. First, this allowed us to evaluate whether the drying behavior and resulting granule quality obtained with ConsiGma-1 was predictive for the ConsiGma-25. Secondly, the effect of drying settings on granule size could also be determined, allowing us to indicate the suboptimal settings that are responsible for excessive breakage of granules, whereby the generation of a large number of fine particles could negatively impact tableting (due to segregation or poor flowability).

## 2. Materials and Methods

### 2.1. Materials

The formulation under study consisted of a low drug-loaded, poorly soluble, and poorly wettable API (BCS class II), a large content of α-lactose monohydrate (Pharmatose 200, DFE Pharma, Goch, Germany), microcrystalline cellulose (MCC; Avicel PH101, FMC biopolymer, Philadelphia, PA, USA), hydroxypropyl methylcellulose (HPMC; Methocel E5, Dow, Midland, MI, USA), croscarmellose sodium (Ac-Di-Sol, FMC, Philadelphia, Pennsylvania, MI, USA) and sodium dodecyl sulphate (Kolliphor SLS, BASF, Ludwighafen, Germany).

### 2.2. Methods

#### 2.2.1. Equipment

**Figure 1:**
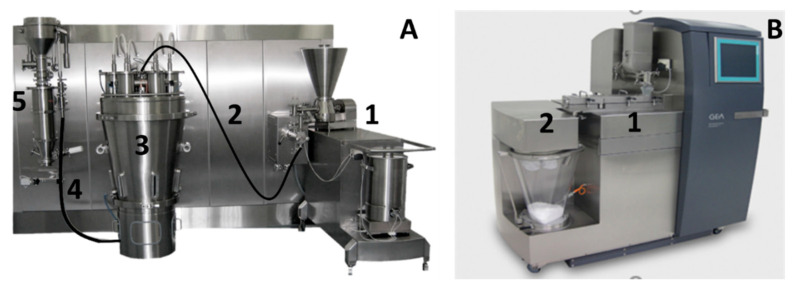
ConsiGma-25 line (**A**): twin-screw wet granulation module (1), wet transfer line (2), six-segmented fluid bed dryer (3), dry transfer line (4), and conditioning unit with mill (5). Laboratory ConsiGma-1 unit (**B**): twin-screw wet granulation module (1) and one-segmented fluid bed dryer (2).

In this study, a horizontal ConsiGma-25 system (C25) (GEA Pharma Systems, Wommelgem, Belgium) and a ConsiGma-1 system (C1) (GEA Pharma Systems, Wommelgem, Belgium) were used. The former is a continuous manufacturing line consisting of a twin-screw wet granulation module, a six-segmented fluid bed dryer, and a granule-conditioning unit with a mill (Figure 1A), as previously discussed in detail by several authors [24,29,30]. This ConsiGma-25 line can also be connected to a tablet press. The ConsiGma-1, on the other hand, is a mobile laboratory unit consisting of a twin-screw wet granulation module (identical to the granulation module of C25) and a single fluid bed dryer (Figure 1B). The drying module of the C1 has the same design as one drying segment of the six-segmented dryer of the C25 system.

#### 2.2.2. Granulation Module

First, a 20 L tumbling blender (Inversina-Bioengineering, Wald, Switzerland) was used for 15 min at 25 rpm to prepare the formulation pre-blend. Subsequently, the pre-blend was transferred to the gravimetric loss-in-weight feeder of the C25 (KT20, K-Tron Soder, Niederlenz, Switzerland) or the C1 (Brabender DDSR20, Duisburg, Germany). Thereafter, the powder mixture was fed into the granulator. For both systems, the granulator was identical and consisted of two 25 mm diameter co-rotating screws with a length-to-diameter (L/D) ratio of 20:1. Demineralized water was used as granulation liquid and was gravimetrically dosed into the granulator using 2 out-of-phase peristaltic pumps (Watson Marlow, Cornwall, UK). Silicon tubing with an internal and external diameter of 1.6 and 4.0 mm, respectively, was connected to 1.6 mm nozzles. The screw configuration was composed of 2 kneading zones of 6 kneading elements (L/D = 1/4) and 2 small chopper elements (L/D = 1/6), inserted at the end of the screws. All elements were positioned in a forward stagger angle of 60° and separated by conveying elements. The jacket of the granulator barrel was pre-heated and furthermore maintained at a temperature of 25 °C.

Process settings were kept constant during all experiments. A mass feed rate (MFR) of 10 kg/h, a screw speed of 675 rpm, and a liquid-to-solid (L/S) ratio of 23% were applied. These settings were chosen on the basis of previous trials (see Section 3 and Section 4) as this resulted in granules with a friability (indication for granule strength) of 22%, a fines fraction of 1.6%, and an oversized fraction of 77.1%. From previous experience, the authors know that processing granules with a friability of 22% allows for the observation of breakage in the system, yet without the creation of an excessive number of fines. The latter could result in clogging of the drying filters.

#### 2.2.3. Fluid Bed Dryer

**Figure 2:**
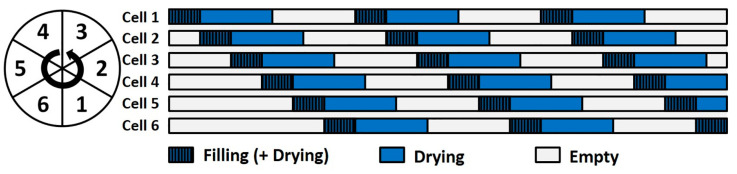
Schematic overview of the different phases during drying in the six-segmented fluid bed dryer of a ConsiGma-25 line. Reproduced with permission from ([29]), Elsevier, 2018.

For the C25, wet granules were pneumatically transferred from the granulator outlet to the six-segmented fluid bed dryer via the wet transfer line. The cells were consecutively filled and emptied in the same order. While one cell was filled for a set filling time, another was drying, discharging, or remained empty, as schematically shown in Figure 2. After completing a drying cycle in a cell, the rotating discharge valve allowed us to pneumatically transport the granules to the granule conditioning unit. To avoid an effect of the start-up phase of the drying process, we only collected samples when the number of operational cells (i.e., number of cells that are simultaneously being filled or dried) was constant. The number of operational cells may vary as this depends on the set drying time. The drying cell of the C1 was gravimetrically filled since the granulator outlet is located on top of the drying cell inlet. Granules were manually collected after the drying process. For all experiments, an identical filling time of 120 s was applied in both equipment, resulting in a nominal cell load of ±333 g. The drying air flow was set at 300 m^3^/h or 50 m^3^/h for the C25 and C1, respectively, as this resulted in an adequate fluidization in both systems. For all experiments, the drying air temperature was preheated to the desired temperature of 40 or 60 °C before the start of an experiment.

#### 2.2.4. Investigating the Particle Size Evolution along the ConsiGma-25 Line

**Figure 3:**
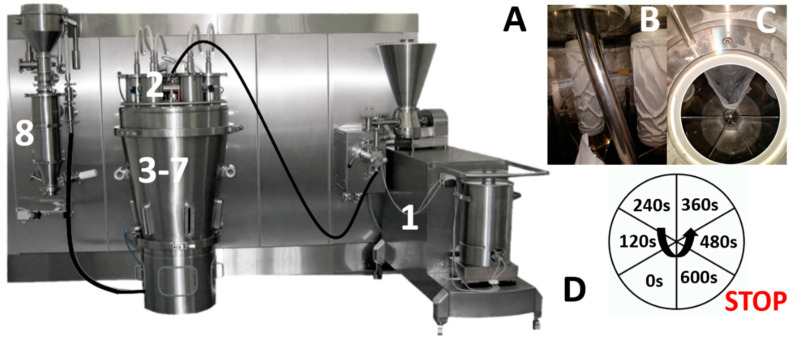
Overview of sample collection at different locations along the ConsiGma-25 line: (**A**) after the granulation module (1), after the wet transfer line (2), at different time points during drying (3–7), and after the conditioning unit (8). Tube (**B**) dividing the granules over the different cells transfers granules directly in a bag (**C**). Schematic overview of drying time corresponding to each cell when process was stopped (**D**).

The evolution of the granule size along the C25 line was evaluated, i.e., between the granulator outlet and the condition unit. For this experiment, an overall drying time of 600 s (including the filling time of 120 s) was applied, while the drying air temperature was set at 40 °C. Granules were collected at 8 different locations to evaluate the granule size (Figure 3A):

- Location 1: After the granulation module. Only the granulation module was used, and granules were subsequently collected at the end of the granulation barrel.
- Location 2: After the wet transfer line connecting the granulator outlet and dryer inlet. A plastic bag was installed in 1 cell (Figure 3B,C) in order to gently collect the granules coming out of the wet transfer line. Once the filling phase was completed, the drying process was stopped, and granules were manually removed from the bag.
- Location 3–7: In the drying cells 1–5: After completing a full drying cycle (i.e., filling, drying, and emptying of cells 1–6), the process was stopped during the second cycle just before the emptying of cell 1 (Figure 3D). Consequently, cells 1, 2, 3, and 4 corresponded with a drying time of 600, 480, 360, and 240 s, respectively. Cell 5 (120 s) thus corresponded to a complete filling phase only.
- Location 8: After the conditioning unit (no milled was installed). Granules subjected to a complete drying process were transferred via the dry transfer line and collected after the conditioning unit.

For all samples, the overall moisture content was evaluated using part of the produced samples. The remainder of each sample served for granule size characterization (Section 2.2.6), for which it was oven-dried (40 °C, 25% relative humidity) until a moisture content of 1.5–2.5% was obtained.

#### 2.2.5. Investigating the Influence of Dryer Settings on Granules’ Critical Quality Attributes (CQAs) for C1 and C25

Drying time and drying air temperature were varied to establish a drying profile for different granule size fractions. In addition, the influence of the drying time and drying air temperature on granule size was investigated. For both systems, drying time was varied at 4 levels, whereas 2 levels of the drying air temperature (40 or 60 °C) were studied. A total of 8 experiments were performed per equipment (Table 1).

Additionally, granules were also produced using only the granulation module. These granules were collected at the outlet and intensively oven-dried at 70 °C. Samples were removed from the oven in function of time and, subsequently, their overall moisture content was determined. These granules were then subjected to a friability test to investigate the influence of moisture content on breakage behavior.

#### 2.2.6. Granule Characterization

##### Granule Size Distribution

Dynamic image analysis (QICPIC, Sympatec, Etten-Leur, the Netherlands) was performed to evaluate the granule size distribution (GSD) of the dried granules. A representative sample of 80 g of granules was fed by a vibratory feeder towards a gravimetric feed tube where the granules were dispersed in front of the measurement window. Volume size distributions were calculated by the WINDOX 5 software (Sympatec, Etten-Leur, The Netherlands). Measurements were performed in duplicate. The size fraction smaller than 150 µm was defined as the fines fraction. On the other hand, the fraction larger than 1000 µm corresponded to oversized granules.

##### Residual Moisture Content

The overall residual moisture content and the residual moisture content per sieved granule fraction (i.e., >2000, 1000–2000, 850–500, 150–500, and <150 µm) was determined via loss-on-drying (LOD) using a Mettler LP16 moisture analyzer (Mettler-Toledo, Zaventem, Belgium). A sample of approximately 3 g was dried at 105 °C until its weight was constant for 30 s. At this point, the percentage LOD was recorded. Measurements were performed in triplicate.

##### Friability

Granule friability was measured to determine granule strength using a friabilator (Pharmatest PTF E, Hainburg, Germany). Before each measurement, granules were pre-sieved at 250 µm. Subsequently, 10 g (Iw) of the fraction larger than 250 µm was combined with 200 glass beads (4 mm diameter) and subjected to 250 rotations for 10 min. Then, the fraction smaller than 250 µm was again removed and the remaining mass (Fw) was weighted. Friability was calculated using Equation (1). Experiments were performed in duplicate.

(1) $$\mathrm{Friability}\;\left(\%\right)=100\;\times \;\frac{\left(\mathrm{Iw}-\mathrm{Fw}\right)}{\mathrm{Iw}}$$

## 3. Results and Discussion

### 3.1. Granule Size Evolution along the Length of the ConsiGma-25

**Figure 4:**
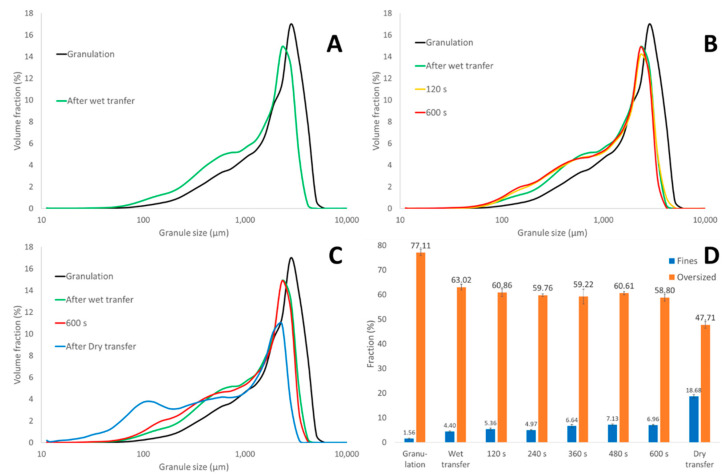
Granule size distributions to study the effect of the transfer via the wet transfer line (**A**), the drying process (**B**), and the transfer of the dried granules via the dry transfer line (**C**). The fraction in fines and oversized granules is shown at different drying times and along the length of the C25 (**D**).

The aim of this first part of the study was to evaluate the breakage and attrition phenomena at different locations along the length of the ConsiGma-25 to have an enhanced process understanding. Granule size distributions obtained at the different locations and their corresponding fractions of fines (<150 µm) and oversized granules (>1000 µm) are shown in Figure 4. Figure 4A illustrates that the wet transfer line caused breakage and attrition to the wet granules. Since crystallization of solubilized powder did not take place before and during this transfer, solids bonds were not yet formed, making granules prone to breakage and attrition. Hence, the fraction of oversized granules decreased from 77.11 to 63.02%, while the number of fines increased from 1.56 to 4.40%. De Leersnyder et al. [29] also reported breakage caused by the wet transfer while comparing a horizontal and vertical set-up of the ConsiGma-25, albeit to a greater extent. In their study, a decrease in oversized granules of 40–60% and an increase in fines from 4 to 8% was observed. The variation in the extent of breakage was possibly due to differences in binder potency, raw material properties such as solubility, and granulation process parameters such as the L/S ratio. Although it is not straightforward to compare different formulations manufactured under different process settings, weaker granules were presumably obtained as a lower L/S ratio was used to granulate a more hydrophobic formulation consisting of two insoluble fillers (maize starch and powdered cellulose). This indicates that formulation and process optimization could minimize breakage in the wet transfer line.

The impact of the filling phase (120 s drying time) as well as the fluid bed drying process (600 s) on the granule size is illustrated in Figure 4B. During the filling phase, the granules entered the cell at high velocity, thereby colliding with the bottom or wall. The first granules were subjected to a turbulent flow due to the limited cell load, enhancing particle–particle and particle–cell wall collisions. As more granules entered the dryer cell, the wetted mass became heavier and the entering granule flow was more stable. Therefore, only a very small shift in GSD and a small change in fines from 4.4 to 5.4% and oversized granules from 63.0 to 60.8% was observed after the filling phase. Furthermore, granule breakage and attrition inside the drying cell was limited during drying. The change in GSD inside the cell after 120 and 600 s drying was small (Figure 4B). Once the cells were filled, the decrease in oversized granules (60.8 to 58.8%) and the increase in fines fraction after 600 s of drying (5.4 to 7.0%) was very moderate. In contrast, the impact of the dry transfer line connecting the dryer cell outlet with the conditioning unit was severe (Figure 4C). Even though the granules were sufficiently dried (LOD of 1–3%) and solid bonds were formed, the powerful vacuum transport and the accompanying blowbacks to completely empty the cell had a large impact on the granule size—the oversized fraction decreased (58.8 to 47.7%), the fines fraction increased (7.00 to 18.7), and a bimodal distribution was obtained (Figure 4C). The change in the fines and oversized fraction at different drying times and along the length of the C25 is shown in Figure 4D.

The applied methodology could determine the initial size distribution of granules entering the cell as well as the change in GSD during drying. As particle size has a great influence on the drying rate [29], the obtained results are important in the development of a generic drying model. Moreover, the results are needed for the development of a general flowsheet model focusing on granule size. A flowsheet model combines several unit operation models to ultimately simulate the final granule size distribution. Van Hauwermeiren et al. [23] presented a first step towards a generalized mechanistic population balance model (PBM) for the prediction of granule size obtained via the twin-screw granulation unit. This study supports this approach as it improves our understanding of the dynamic behavior of the granule size along the other units of the manufacturing line.

### 3.2. Influence of Dryer Parameters on Granule CQAs Per Equipment

#### 3.2.1. Moisture Content

**Figure 5:**
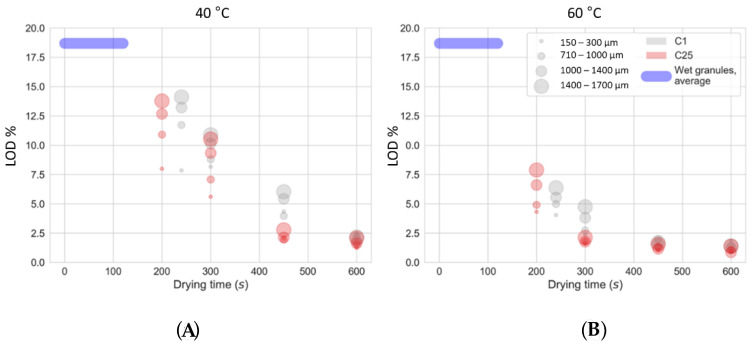
Residual moisture content (LOD values) of the granules produced on C1 and C25 in function of drying time at 40 °C (**A**) and 60 °C (**B**) for different size fractions (smaller size fractions are represented by smaller dots, whereas larger size fractions are represented by larger dots). The average moisture content of granules entering the drying cells during the filling phase was depicted by the blue bar.

In this section, the impact of drying air temperature and drying time on moisture content is evaluated and compared for granules processed with C1 and C25 (Table 1). The granule drying profiles per granule size fraction at 40 and 60 °C are illustrated in Figure 5. During the complete filling phase, granules with an overall moisture content of 18.7% were introduced into the cells on the basis of the powder and liquid feeding rate. Generally, a fast water evaporation occurs at the surface of the granules in the first drying phase, resulting in a fast decline of moisture content. Hereafter, intra-granular liquid evaporation starts during the second drying phase and the produced vapor is transferred through the granule pores towards the surface. Hence, the second drying phase is slower and finally ends when a similar and targeted residual moisture content (1.0–2.5%) is reached for all size fractions. This two-phase drying trajectory indicates that a sufficient drying time needs to be applied to reach a uniform drying of the particles. At that moment, all particles—independent of granule size—are in equilibrium with the drying medium [31].

Larger granules dried slower than smaller size fractions for three reasons. First, as in accordance with the results of De Leersnyder et al. [29], the surface-to-volume ratio is lower for larger granules. Secondly, larger granules contain more liquid since heterogeneous powder–granulation liquid mixing is realized during twin-screw granulation [7], and hence, more time is needed to remove the liquid. Further, intra-granular vapor has to traverse a larger length as the distance between granule core and surface is larger. Figure 5 also shows that a high drying temperature strongly affects the drying trajectory, whereby a fast initial decline in moisture content was obtained at a high temperature. However, even at 60 °C, the moisture content even further slightly decreased from 450 to 600 s, showing that the equilibrium is reached slowly.

In this study, a fixed filling time of 120 s was used. Therefore, the number of operational cells while applying a drying time of 300, 450, and 600 s was 3, 4, and 5 on the C25, respectively. Since the resistance to the air flow is higher in filled cells, the drying air was expected to preferentially pass through empty cells [28]. Consequently, a more efficient drying was expected on C1 because the equipment consisted only of a single cell. However, the second drying phase was delayed for granules processed on the C1, as a plateau phase only started after 450 and 600 s for a drying temperature of 60 and 40 °C, respectively, whereas this was already seen after 300 and 450 s when drying in the C25. The lower moisture content in the C25 compared to the C1 can be explained by the transfer of heat by convection and conduction from the surrounding empty cells to the cells filled with granules. A similar phenomenon is impossible on the C1 and a higher moisture content is therefore obtained. Similar results were observed by Stauffer et al. and Vercruysse et al. [25,28]. In addition, it is possible that the pneumatic transport on the C25 can provide additional evaporation of the liquid on the granule surface. As previously mentioned, this study could help in the development of a generic drying model. Ghijs et al. [32] already modelled the drying behavior, at a constant material flow rate and filling time and with respect to granule size, in the drying-unit of the vertical ConsiGma-25 line. Since the moisture content per size fraction was determined in this study, this study could also serve to improve the calibration of the mechanistic drying model.

#### 3.2.2. Granule Size

##### ConsiGma-25

**Figure 6:**
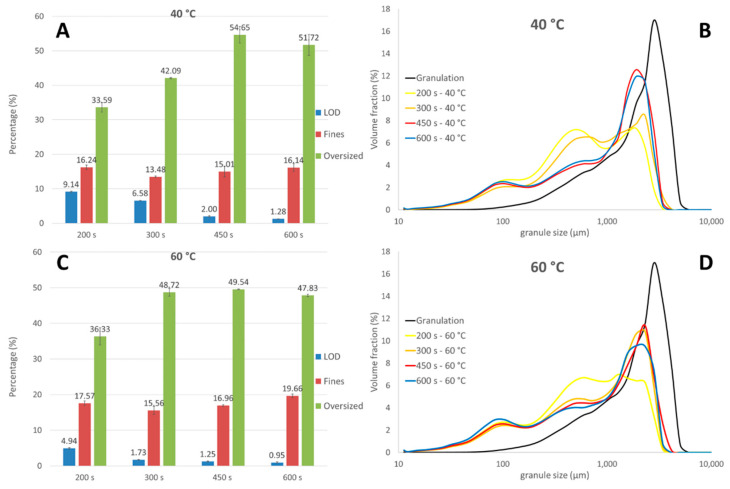
The fraction in fines and oversized granules and the corresponding overall moisture content after different drying times for granules collected after the conditioning unit at 40 (**A**) and 60 °C (**C**). Granules size distributions to visualize the effect of drying time at 40 (**B**) and 60 °C (**D**).

In this section, the effect of drying process settings on the final granule size (i.e., granules collected after the dry transfer line) was evaluated. From Section 3.1, it is clear that breakage and attrition was very limited during drying. In addition, the degree of breakage and attrition in the wet transfer line was assumed to be identical for each experiment (Figure 4A) as the granulation process was robust. Hence, the investigation of the effect of drying parameters on particle size of granules processed on the C25 mainly corresponded to investigating the effect of drying parameters on the breakage in the dry transfer line. Figure 6 illustrates the overall moisture content and the fraction of fines and oversized granules for each experiment (Table 1) and, in addition, the corresponding GSD. Generally, the extent of breakage and attrition caused by the dry transfer line depended on the residual moisture content of the granules. A higher moisture content due to insufficient drying resulted in more breakage in the dry transfer line as less solid bonds were formed. This was demonstrated for granules that were only dried for 200 or 300 s at a drying temperature of 40 °C. Fewer oversized granules and more fines were observed as these granules still contained 9.1 and 6.6% moisture, respectively. A nearly identical GSD was observed for 450 and 600 s of drying, indicating a similar breakage behavior, as these granules had a similar residual moisture content, i.e., only 1–2%. A drying temperature of 60 °C resulted in a more pronounced breakage only at the lowest drying time—with a higher number of fines and lower amount of oversized granules—presumably because the overall moisture content was still higher (i.e., 4.9%). As a moisture content between 1 and 2% was observed for granules dried for 300 and 450 s, an almost identical GSD was measured. Surprisingly, granules were further broken to a greater extent when the most extreme drying condition —600 s at 60 °C—was used, resulting in slightly more fines (19.6%) and less oversized granules (47.8%). These granules contained less than 1% moisture.

**Figure 7:**
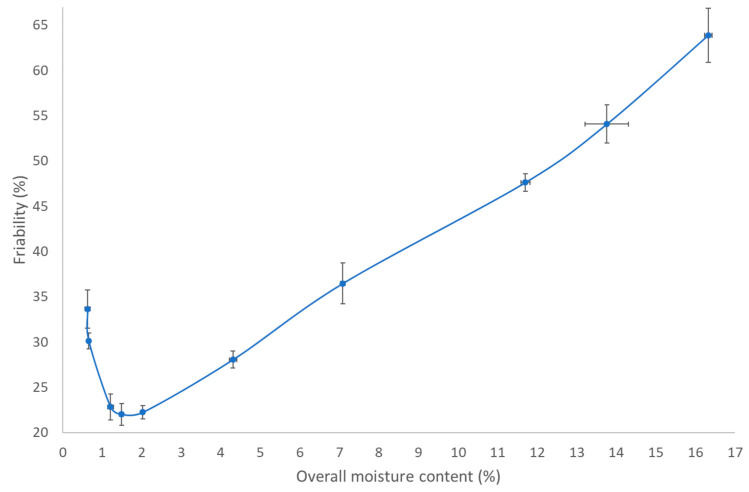
Friability as a function of overall residual moisture content.

To understand these phenomena, we performed an additional experiment to illustrate the effect of granule residual moisture content on granule strength. For this experiment, granules obtained after the granulation module were intensively oven-dried at 70 °C. Figure 7 depicts the overall residual moisture content in function of friability (i.e., a measure for granule strength). Friability decreased at lower moisture content because more solid bridges were formed when liquid was evaporated from the granules. The lowest friability was obtained for granules having a residual moisture content between 1 and 2.5%. However, further liquid elimination weakened the granules again, indicating that a residual moisture of 1–2.5% in granules provided some plasticity to endure mechanical stress. This is in accordance with the statements of Mezhericher et al. [31], where the author stated that drying-induced stress occurred more at higher temperature. At higher temperature, an accumulation of vapor might occur in the particle core during the second drying stage as the excess of vapor can possibly not easily escape through the pores towards the surface. This leads to a higher internal temperature and pressure gradient that might damage the granule microstructure, making the granules prone to mechanical stress, which leads to more breakage [33]. Hence, the increase in friability at residual moisture below 1% can be explained since more liquid was evaporated and granules were subjected for a longer time to a high temperature (70 °C was applied to induce an intensive drying), and a higher temperature and pressure gradient was obtained, making the granules more prone to breakage. Evidently, this explains the additional breakage at 60 °C and 600 s, as seen in Figure 6.

**Figure 8:**
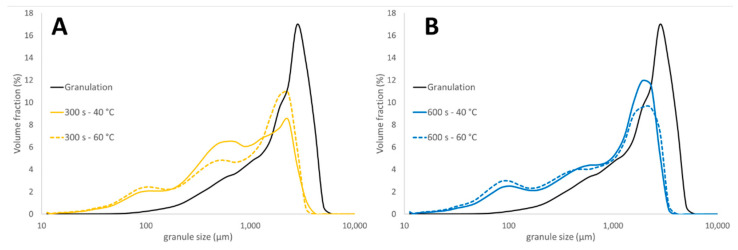
Granule size distribution to study the effect of drying air temperature on granule size after 300 (**A**) and 600 s (**B**) of drying.

De Leersnyder et al. [29] stated that drying air temperature had no effect on granule size. Conversely, in this study, a clear difference in GSD for granules dried at 40 and 60 °C was observed via a head-to-head comparison. On the one hand, a drying air temperature of 40 °C resulted in more breakage compared to a drying air temperature of 60 °C for the same drying time of 300 s. This could have been due to an insufficient drying at 40 °C within this period of 300 s, as explained in the beginning of the section (Figure 8A). On the other hand, even more breakage was observed with a drying air temperature of 60 °C after an extended drying time of 600 s, as the resulting very dry granules (<1 moisture content) were more prone to breakage (cf. breakage behavior observed in Figure 7). This demonstrated that the final particle size primarily depended on the moisture content obtained after drying and, consequently, the final particle size was indirectly governed by the drying parameters. De Leersnyder et al. [29] compared the final GSD (collected after the conditioning unit) for granules processed with a horizontal and a vertical set-up. The vertical set-up also showed extensive breakage, although it was to a lesser extent compared to the horizontal set-up due to the absence of the wet transfer line, indicating that the breakage caused by the dry transfer line was present for both forms of equipment. This emphasizes that an optimal drying process, granulation process, and formulation need to be developed to minimize the breakage and attrition phenomena.

##### ConsiGma-1

**Figure 9:**
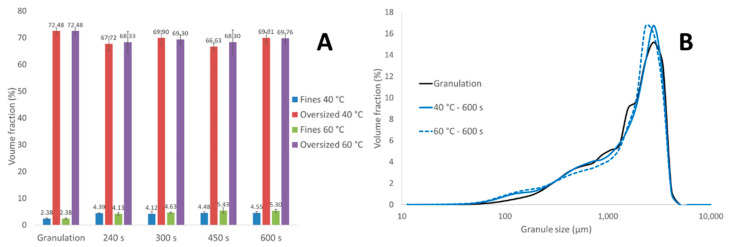
The fraction of fines and oversized granules after granulation and at different drying time points on the C1 (**A**). Granule size distribution after 600 s of drying at 40 and 60 °C of drying air temperature (**B**).

Granules were directly transferred by gravitation from the granulation module to the drying cell at the C1 equipment (Figure 1). Breakage due to the wet transfer line was therefore absent. Only a moderate change in the fraction in fines and oversized granules was observed after 240 s of drying (Figure 9A), indicating that minor breakage and attrition occurred during the filling phase, similar to the filling phase at C25. By comparing the fines and oversized fraction at different time points at 40 and 60 °C, we found that drying air temperature and drying time did not have any relevant influence on granule size. This was also seen by examining the GSD after 600 s drying at 40 and 60 °C, as a nearly identical GSD was observed. Similar to the drying process with the C25, minimal breakage and attrition phenomena were observed during the drying process.

As granules were manually removed from the drying cell, the impact of the dry transfer line was also missing. Therefore, a completely different GSD was seen at the end of the process between granules from C1 (Figure 9) and C25 (Figure 6). The unimodal GSD from the C1 was not comparable with the bimodal GSD from the C25. During formulation development and process optimization, the final GSD obtained on the C25 equipment could therefore not be estimated on the basis of the C1.

## 4. Conclusions

In this study, it was possible to elucidate where and to what extent breakage and attrition occurred along the length of the C25 line. It was shown that breakage occurred in the wet transfer line because solid intra-granular bonds were not yet formed. Furthermore, breakage and attrition during drying were very limited, whereas the pneumatic transfer via the dry transfer line caused extensive breakage as the drying cells were powerfully emptied. Since fixed granulation parameters were applied on the same formulation, the extent of breakage presumably differs when different parameters and different raw materials are applied. However, it is suggested that similar breakage and attrition phenomena would occur at the studied locations, regardless of the formulation.

This study showed that the moisture content influenced the final granule size on C25, as it co-determines granule strength. It was found that an optimal moisture content range for granule strength exists, and granules were found to be more prone to breakage when having a moisture content below and above the range of 1–3% due to drying-induced thermal stresses and incomplete solid bond formation, respectively. Moreover, the current study also demonstrated that the residual moisture content was generally lower for identical process parameters for granules processed on C25 because heat was transferred by convection and conduction from the surrounding empty cells. The final particle size of granules processed on C1 differed substantially, making it difficult during process development to obtain a granule quality that is representative for the C25. Overall, this study provided an enhanced understanding in the breakage behavior during fluid bed drying, which is important in process and formulation optimization and process control. Further, the obtained results are useful in the development of a generic drying model or a flowsheet model used for the simulation of granule size.

## Figures and Tables

**Table 1:** Overview of the applied drying parameters.

Experiment Number	Drying Time (s) C1	Drying Time (s) C25	Drying Temperature (°C)
1	240	200	40
2	300	300	40
3	450	450	40
4	600	600	40
5	240	200	60
6	300	300	60
7	450	450	60
8	600	600	60

## Data Availability

Not applicable.
